# Metatranscriptomic Analysis Reveals Synergistic Activities of Comammox and Anammox Bacteria in Full-Scale Attached Growth Nitrogen Removal System

**DOI:** 10.1021/acs.est.4c04375

**Published:** 2024-07-13

**Authors:** Juliet Johnston, Katherine Vilardi, Irmarie Cotto, Ashwin Sudarshan, Kaiqin Bian, Stephanie Klaus, Megan Bachmann, Mike Parsons, Christopher Wilson, Charles Bott, Ameet Pinto

**Affiliations:** † School of Civil and Environmental Engineering, Georgia Institute of Technology, Atlanta, Georgia 30332, United States; ‡ Department of Civil and Environmental Engineering, Northeastern University, Boston, Massachusetts 02115, United States; § Department of Environmental and Occupational Health Sciences, University of Washington, Seattle, Washington 98195, United States; ∥ Hampton Roads Sanitation District, Virginia Beach, Virginia 23455, United States; ⊥ Department of Civil and Environmental Engineering, Virginia Tech, Blacksburg, Virginia 24061, United States

**Keywords:** comammox−anammox cooperation, metatranscriptomics, IFAS, wastewater

## Abstract

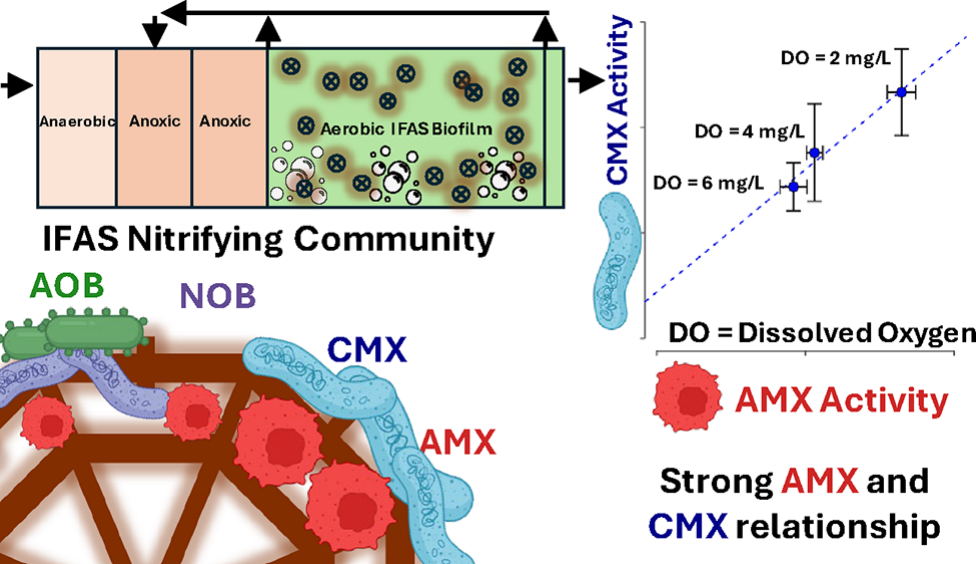

Leveraging comammox *Nitrospira* and anammox bacteria for shortcut nitrogen removal can drastically lower the carbon footprint of wastewater treatment facilities by decreasing aeration energy, carbon, alkalinity, and tank volume requirements while also potentially reducing nitrous oxide emissions. However, their co-occurrence as dominant nitrifying bacteria is rarely reported in full-scale wastewater treatment. As a result, there is a poor understanding of how operational parameters, in particular, dissolved oxygen, impact their activity and synergistic behavior. Here, we report the impact of dissolved oxygen concentration (DO = 2, 4, 6 mg/L) on the microbial community’s transcriptomic expression in a full-scale integrated fixed film activated sludge (IFAS) municipal wastewater treatment facility where nitrogen removal is predominantly performed by comammox *Nitrospira* and anammox bacterial populations. 16S rRNA transcript compositions revealed anammox bacteria and *Nitrospira* were significantly more active in IFAS biofilms compared to suspended sludge biomass. In IFAS biofilms, anammox bacteria significantly increased *hzo* expression at lower dissolved oxygen concentrations and this increase was highly correlated with the *amoA* expression levels of comammox bacteria. Interestingly, the genes involved in nitrite oxidation by comammox bacteria were significantly more upregulated, relative to the genes involved in ammonia oxidation with decreasing dissolved oxygen concentrations. Ultimately, our findings suggest that comammox *Nitrospira* supplies anammox bacteria with nitrite via ammonia oxidation and that this synergistic behavior is dependent on dissolved oxygen concentrations.

## Abstract 2

Comammox bacteria differentially regulate ammonia and nitrite oxidation in response to dissolved oxygen concentration suggesting dissolved oxygen dependence of their synergistic nitrogen removal with anammox bacteria in IFAS biofilms.

## 1 Introduction

Recent studies have suggested that cooperation between comammox bacteria (CMX) and anammox bacteria (AMX) can successfully result in nitrogen removal from mainstream wastewater in both laboratory^1−3^ and full-scale systems.^4^ Typical full-scale systems have nitrifying communities often pairing strict ammonia oxidizing bacteria (AOB) with nitrite oxidizing bacteria (NOB), AOB with AMX, or a combination of AOB, CMX, and NOB for nitrogen removal.^5^ However, such cooperation between CMX and AMX could result in nitrogen removal with reduced oxygen and carbon demand, lower carbon, alkalinity, and tank volume requirements while also significantly reducing biotic production of nitrous oxide (N_2_O),^6,7^ which is a highly potent greenhouse gas (GHG). This contrasts with conventional nitrogen removal systems, where estimates suggest that 1–1.5% of incoming nitrogen is lost as fugitive N_2_O emissions.^8^ Thus, the cooperation of CMX and AMX for wastewater treatment has the potential to significantly decarbonize wastewater treatment.

While CMX are found in soils,^9−11^ drinking water systems,^12−14^ and traditional activated sludge systems,^15−18^ they are far more likely to prefer biofilms in attached growth systems and systems with long solids retention time (SRT)^19^ due to their slow growth rates.^20,21^ Similarly, AMX may also prefer biofilms and granules due to their slower growth rates and because such aggregate environments may offer protection from their sensitivity to nitrite inhibition in nitrogen-rich wastewater sources^22,23^ while also preventing exposure to dissolved oxygen (DO).^24,25^ The cohabitation of CMX and AMX in biofilms provides an ideal scenario to drive nitrogen loss via their metabolic coupling, especially in comparison to strict AOB. For instance, recent long-term studies have demonstrated nitrifier denitrification mediated by AOB as the primary source of N_2_O emissions under oxygen-limiting conditions,^26^ conditions that are essential for robust AMX activity.^27,28^ Thus, a CMX–AMX-driven nitrogen removal process has the potential to significantly reduce GHG emissions. Further, there is potential for CMX to resupply AMX with nitrite from nitrate under anaerobic conditions since the activity of the nitrite oxidoreductase enzyme has been demonstrated to be reversible. Nitrate reduction has been shown in other *Nitrospira* bacteria using formate and hydrogen.^29^ Similarly, comammox *Nitrospira* have been shown to have formate dehydrogenase^29,30^ and hydrogenases,^31^ suggesting the same potential. This nitrate may be due to complete nitrification or residual nitrate produced due to AMX activity. Thus, a CMX-AMX process has the theoretical potential to deliver a single-unit process with complete nitrogen removal, where even residual levels of nitrate produced by the anammox metabolism and any nitrate produced by comammox can be completely removed.

Our previous work identified CMX and AMX as dominant nitrifying bacteria in biofilms in a full-scale integrated fixed film activated sludge (IFAS) process;^4^ this was the first report to observe their potential cooperation in a full-scale municipal wastewater treatment system, while similar studies have struggled to maintain both CMX and AMX activity when attempting bioaugmentation.^32^ In this study, we observed that anaerobic ammonia oxidation decreased while aerobic ammonia oxidation to nitrate increased with increasing DO concentration. In the IFAS biofilms, two AMX bacterial populations were associated with *Ca.* Brocadia, comprising 6.53 ± 0.34% of the population, while CMX bacteria were closely associated with *Ca. Nitrospira* nitrosa, comprising 0.37 ± 0.03% of the population. This system still had an abundance of strict AOB and nitrite-oxidizing bacteria (NOB), particularly in the suspended phase, making it unclear the extent to which organisms were likely supplying nitrite to AMX.^19^

The current study follows up our previous metagenomic and kinetic investigation by systematically characterizing metatranscriptomic changes of the microbial communities in the suspended phase and IFAS biofilms in response to changing DO concentrations. To do this, we combine genome-centric metatranscriptomics as well as amplicon sequencing and quantitation of major functional gene expressions (*amoA* for AOB, *amoA* for CMX, *nxrB* for *Nitrospira* bacteria, which includes strict NOB and CMX, and *hzo* for AMX) to determine (1) potential metabolic coupling between different nitrifying populations within this mixed community and (2) transcriptomic responses of key nitrifying populations as a function of DO concentrations and growth mode (i.e., attached vs suspended phase). We systematically explore how the entire microbial community is impacted by dissolved oxygen then focus on all nitrifying bacteria and their unique interactions. CMX and AMX are the primary focus since no other full-scale treatment facility has shown this combination of nitrifying bacteria as the two dominant sources of nitrogen removal. By understanding the activity of all nitrifying organisms (AOB, CMX, NOB, AMX) in a complex integrated fixed film activated sludge (IFAS) wastewater treatment facility, we can identify DO concentrations that produce the highest activity and potential synergy between CMX and AMX. Such insights have the potential to advance the development of mainstream partial nitritation-anammox nitrogen removal systems with lower energy requirements and GHG emissions.

## 2 Materials and Methods

### 2.1 Overview of Experimental Design

A detailed overview of the wastewater treatment facility’s process flow diagrams, operational parameters, and conditions is reported in Vilardi et al.^4^ In brief, the secondary treatment consists of five zones in series including an anaerobic stage followed by an anoxic stage receiving internal nitrate recycle divided into two cells, an aerobic stage with IFAS, and a small deaeration zone. Samples were taken at the anaerobic stage (R1), the end of the anoxic zone (R3), and twice within the aerobic IFAS stage at the beginning and the end (R4 and R5). Full-scale tank DO concentrations in the aerobic zone were stabilized for 6 h prior to sampling, and experiments for multiple DO set points were run on consecutive days within a week to eliminate potential confounding effects from changes in microbial community composition. Samples for DNA-based analyses were immediately stored in dry ice, while samples for RNA-based analyses were mixed at a 1:10 LifeGuard Soil Preservation (Qiagen, Hilden, Germany) before being put on dry ice. At the end of each day’s sampling, all samples were stored at −80 °C until extraction.

### 2.2 Nucleic Acid Extraction

DNA was extracted using protocols from the DNeasy PowerSoil Pro Kit with some modifications (Qiagen, Hilden, Germany) as described previously.^4^ Extracted DNA was quantified using a Qubit 4 fluorometer and standard protocols for dsDNA with high sensitivity (ThermoFisher Scientific, Waltham, Massachusetts, USA). RNA was extracted using protocols from the Quick-RNA fecal/soil microbe microprep kit with some modifications (Zymo, Irvine, California, USA). RNA samples were initially preserved with 1 mL of activated sludge (i.e., suspended phase) in 9 mL of LifeGuard soil preservation (Qiagen, Hilden, Germany). This solution was thawed and centrifuged (16,000*g* for 1 min) to pellet the activated sludge. The 1 mL of activated sludge was then transferred into a 2 mL microcentrifuge tube, and 1 mL of ultrapure DNase/RNase-free water was added. To remove residual RNA stabilization solutions, three rinses were performed, where the samples were vortexed for 30 s and centrifuged for 1 min at 16,000*g*, and 1.5 mL of supernatant was exchanged. IFAS samples were stored in 9 mL of LifeGuard soil preservation (Qiagen, Hilden, Germany). Each piece of IFAS media was weighed before and after biomass removal to measure the biomass concentrations for downstream protocols. To remove the biomass from the IFAS plastic, IFAS and LifeGuard soil preservation were transferred into a 5 mL bead beating tube from DNeasy PowerWater kits for 5 min of vortexing (Qiagen, Hilden, Germany). Afterward, 500 μL of biomass and LifeGuard soil preservation were transferred into a clean 2 mL microcentrifuge tube for rinsing to remove the RNA stabilization solution as described above.

Modifications made during the Quick-RNA fecal/soil microbe microprep kit include using a FastPrep-24 homogenizer for 40 s at 6 m/s during bead beating. An additional DNase_1 treatment was performed to degrade residual DNA (Zymo, Irvine, California, USA) followed by the final elution of RNA in 50 μL of buffer. The eluted RNA and residual DNA were quantified via a Qubit fluorometer (ThermoFisher Scientific, Waltham, Massachusetts, USA). After quantification, several aliquots were made where some eluted RNA was immediately frozen at −80 °C for metatranscriptomic sequencing, while another aliquot was processed for cDNA synthesis for RT-qPCR and amplicon sequencing before being frozen at −20 °C.

### 2.4 Quantification of Transcripts and Genes

All samples were quantified for expression, which included duplicate extractions from two technical replicates from all three dissolved oxygen settings, at all reactor locations (R1, R3, R4, and R5 for both suspended sludge biomass and IFAS). For DNA-based gene quantification, only technical duplicates from R1 and R5 for suspended sludge biomass and IFAS were used as there was no significant change in community structure over the short experimental period (i.e., less than 1 week). The extracted RNA was synthesized into cDNA using standard protocols for the SuperScript IV First-Strand synthesis system for RT-PCR (Invitrogen, Waltham, Massachusetts, USA). The cDNA was quantified via a Qubit fluorometer (ThermoFisher Scientific, Waltham, Massachusetts, USA). RT-qPCR and qPCR were then performed on a QuantStudio 7 Flex instrument (ThermoFisher Scientific, Waltham, Massachusetts, USA). The reaction Mastermix contained 0.5 μL of both forward and reverse primers at 10 μM, 10 μL of Luna Universal qPCR Master Mix (New England Biolabs, Ipswich, Massachusetts, USA), 0.5 μL of template DNA/cDNA, and 8.5 μL of nuclease-free water. All primers were purchased from Integrated DNA Technologies (IDT, Coralville, Iowa, USA).

Primers and their thermocycling conditions are shown in Table S1 using standard protocols for 16S rRNA V4 region (515F and 806R),^33^ ammonia monooxygenase subunit A (*amoA*) for strict AOB (amoA-1Fmod and amoA_2R),^34^*amoA* for *Ca.* Nitrospira nitrosa (496F and 812R),^35^ nitrite oxidoreductase subunit B (*nxrB*) for *Nitrospira* bacteria (nxrB169f and nxrB638r),^36^ and hydrazine oxidoreductase (*hzo)* found in AMX (hzocl1F1 and hzocl1F2).^37^ Quantification data of transcripts are shown in Figure S1 with a comparison between reporting RT-qPCR results as log(copies/mL) and log(copies/g of Total Solids) in Figure S2. Since RNA extractions were performed from an aqueous sample (i.e., 1 mL of activated sludge and 500 μL of IFAS media suspended in LifeGuard solution), we present RT-qPCR data as copies/mL. Please also note that we have performed systematic comparisons and found that transcriptomic expression data presented as copies/mL or copies/gram of biomass are essentially identical (see Figure S2).

### 2.5 Amplicon Sequencing

Extracted DNA and cDNA were submitted to Georgia Institute of Technology’s Molecular Evolution Core for sequencing using the Illumina MiSeq v3 kit PE300 using Illumina Nextera Adapter Sequences (Illumina Inc., San Diego, California, USA). The same primers used for RT-qPCR amplification and quantification were fabricated with Nextera adapters for amplicon sequencing to ensure parity with previous similar studies.^38−42^ The samples were demultiplexed, and barcodes were removed by the sequencing core. The raw sequences were then imported into RStudio v2023.06.2^43^ and processed using DADA2 v1.26.^44^ In DADA2, 16S rRNA amplicons used standard protocols with a lower default truncQ = 2. Other amplicons did not use any truncation, and during merging, maxMismatch was increased to 5 to allow longer amplicons to have substantial overlap. Taxonomic assignments referenced the SILVA SSU 138.1 database^45^ and additionally checked in with the MiDAS 5.0 database.^46^ Merged reads outside ±2 bp of the expected amplicon length were discarded. We observed a large number of ASVs post DADA2 processing, which was not congruent with our prior metagenomic analysis.^4^ This could be due to (1) low abundance taxa that are not picked up by metagenomics, (2) residual sequencing error, and (3) natural variation in sequence between functional genes that are often multicopy within bacterial genomes. As a result, ASV’s were further consolidated into OTUs with a 97% identity cutoff using BioStrings v2.66.0,^47^ dplyr v1.1.2,^48^ tibble v3.2.1,^49^ and DECIPHER v2.26.0.^50^ Histograms of post processed amplicon reads are available in the Supporting Information (Figure S3). Amplicon compositions for *amoA* for AOB, *amoA* for CMX, *nxrB* for *Nitrospira* sp., and *hzo* for AMX can be found in Figure S4 with top BLAST matches based on the e-value with the reported sequence percent identity in similarity in Table S2. Absolute quantification of the number of OTUs was estimated by multiplying the relative abundance of the OTU with the number of RT-qPCR copies for each gene.

### 2.6 Metatranscriptomic Sequencing and Data Processing

A limited subset of samples was submitted for metatranscriptomic sequencing in duplicate, limited to only DO concentrations of 2 and 6 mg/L across reactor locations R1, R3, and R5 for both suspended sludge biomass and IFAS. These samples were concurrently collected for metagenomic analysis of this wastewater treatment plant, which has been previously described.^4^ Extracted RNA samples were submitted to the Georgia Institute of Technology’s Molecular Evolution Core for rRNA depletion and RNA sequencing. RNA integrity, read length, and quantification were performed on a 2100 Bioanalyzer with C0.1.069 firmware (Agilent, Santa Clara, California, USA). The minimum RNA integrity number (RIN) used was 6, with an average RIN value of 6.93 ± 0.61 across all samples. Excess rRNA was depleted using the QiaSeq FastSelect 5*S*/16*S*/23S depletion kit as well as the FastSelect HMR and Plant rRNA probes (Qiagen, Hilden, Germany). RNA sequencing was performed on an Illumina NovaSeq S1 PE100bp instrument to obtain 1.6 billion reads (Illumina Inc., San Diego, California, USA). Samples were demultiplexed, and barcodes were removed by the core facilities prior to releasing sequences.

Raw reads were preprocessed using fastp v0.23.4^51^ before residual rRNA reads were separated from mRNA reads using SortMeRNA v2.1.^52^ The remaining mRNA reads were then competitively mapped to previously assembled metagenome-assembled genomes (MAGs) from AOB, CMX, NOB, and AMX.^4,15,19^ The MAGs were annotated using bakta v1.8.2.^53^ The mapped mRNA reads were converted into .bam files, sorted, and indexed using samtools v1.18.^54^ Finally, the mapped mRNA reads were quantified for read counts on the annotated MAGs using dirseq v0.43.^55^

### 2.7 Statistical Methods

Statistical analysis was performed in RStudio^43^ with the vegan package.^56^ ANOVA was used to determine the impact of DO and reactor zonation across all conditions, while Tukey honest significant differences^57^ posthoc test was used to compare means between two subgroups. For the correlative regression analysis, ranked-based linear regressions were performed in R to determine significant trends across RT-qPCR samples. Shannon diversity index^58^ was additionally used to compare the alpha diversity across all amplicon sequencing samples. Differential gene expression analysis was performed with DESeq2.^59^ Within DESeq2, the *p*-values were adjusted using the Benjamini–Hochberg multiple comparison correction. For CMX, the minimum read counts per gene were set to five due to lower transcriptomic reads, whereas the default of 10 read counts was used for AMX. Differential gene expression greater than 2-fold expression and *p*-values of <0.05 were considered significant.

### 2.8 Data Availability

All code for amplicon sequencing, metatranscriptomic analysis, statistical analysis, and plots are available at github.com/queermsfrizzle/CMX_AMX_transcriptomics. Raw fastq files for metatranscriptomic sequencing and amplicon sequencing are available via NCBI bioproject submission number PRJNA1050761.

## 3 Results and Discussion

### 3.1 Microbial Community Composition and Activity Was Associated with Growth Phase and DO concentrations

**Figure 1:**
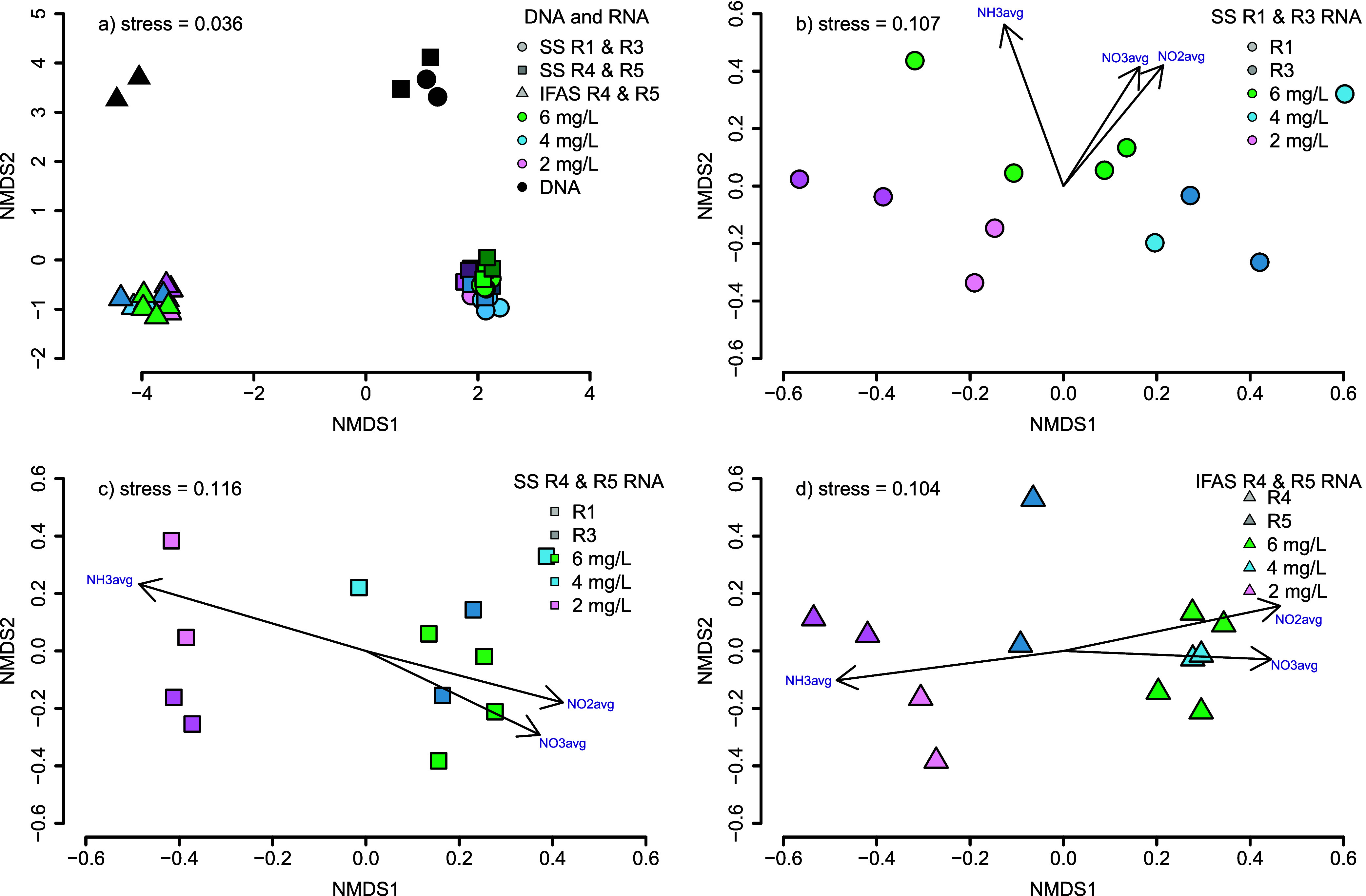
NMDS plots constructed using Bray–Curtis dissimilarity estimates for (a) all 16S rRNA gene and transcript OTUs, (b) SS for R1 and R3 16S rRNA transcript OTUs, (c) SS for R4 and R5 16S rRNA transcript OTUs, and (d) IFAS R4 and R5 16S rRNA transcript OTUs. Black data points represent genes, while colored data points represent transcripts. Green, blue, and pink shades represent the dissolved oxygen concentration at 6, 4, and 2 mg/L, respectively. Shades of each color represent different specific reactor locations, while circles, squares, and triangles represent the reactor zones.

The total and active microbial community compositions were distinct from each other between the two phases (i.e., suspended sludge biomass vs attached IFAS phase) based on 16S rRNA gene and transcript composition (Figure 1). The largest difference in community composition was between IFAS and suspended sludge biomass (Figure 1a), which explained 27.6% of the total variance between samples (PERMANOVA *p* = 0.001, *R*^2^ = 0.99). There was also a significant difference between each community’s 16S rRNA transcript composition and 16S rRNA gene composition, which explained 9.6% of the total variance (Figure 1a) (PERMANOVA *p* = 0.002, *R*^2^ = 0.47). This is consistent with previous reports which showed the most significant changes to community composition based on reactor zonation followed by the separation of DNA and RNA, and further by the experimental variable of seasonality.^38^ When analyzing the Shannon diversity index of 16S rRNA transcripts and genes, there was a significantly higher amount of diversity in 16S rRNA genes, compared to transcripts (ANOVA *p* = 0.0002, *R*^2^ = 0.82) (Figure S4). This suggests that a large proportion of the microbial community is likely less active.^60^

For both IFAS biofilms and suspended sludge biomass communities, the DO concentration had a significant impact on the active community composition (PERMANOVA *p*_IFAS_ = 0.0015, *p*_ss_ = 0.0007) (Figure 1b–d). For the IFAS biofilm community, there was a statistically significant difference between DO = 2 mg/L with DO = 4 mg/L as well as DO = 6 mg/L (ANOSIM *p*_IFAS_^D2D4^ = 0.027 and *p*_IFAS_^D2D6^ = 0.003), but not between DO = 4 mg/L and DO = 6 mg/L (ANOSIM *p*_IFAS_^D4D6^ = 0.5687). In suspended sludge biomass, all dissolved oxygen concentrations showed statistically significant separation (ANOSIM *p*_SS_^D2D4^ = 0.0004, *R*^2^ = 0.63, *p*_SS_^D2D6^ = 0.0004, *R*^2^ = 0.58, *p*_SS_^D4D6^ = 0.0127, *R*^2^ = 0.23). While the DO concentrations did result in significant changes, the measured concentrations of DO, ammonia, nitrite, nitrate, alkalinity, and COD all showed statistically significant effects (Mantel_DO_*p* = 0.022, *R*^2^ = 0.32, Mantel_NH3_*p* = 0.0007, *R*^2^ = 0.57, Mantel_NO2_*p* = 0.004, *R*^2^ = 0.44, Mantel_NO3_*p* = 0.027, *R*^2^ = 0.29, Mantel_alk_*p* = 0.004, *R*^2^ = 0.5, Mantel_COD_*p* = 0.002, *R*^2^ = 0.54) on differences between the active community composition between samples from the IFAS media or suspended sludge biomass. This indicates that the DO set point has cascading effects on microbial community activity and redox conditions due to residual oxygen, nitrite, and nitrate. Dissolved oxygen concentration has been shown to drive the redox conditions for the removal of BOD, ammonia, nitrite, and nitrate, which impacts the microbial community composition.^61−63^

### 3.2 High Diversity of NOB Compared to Strict AOB and AMX

**Figure 2:**
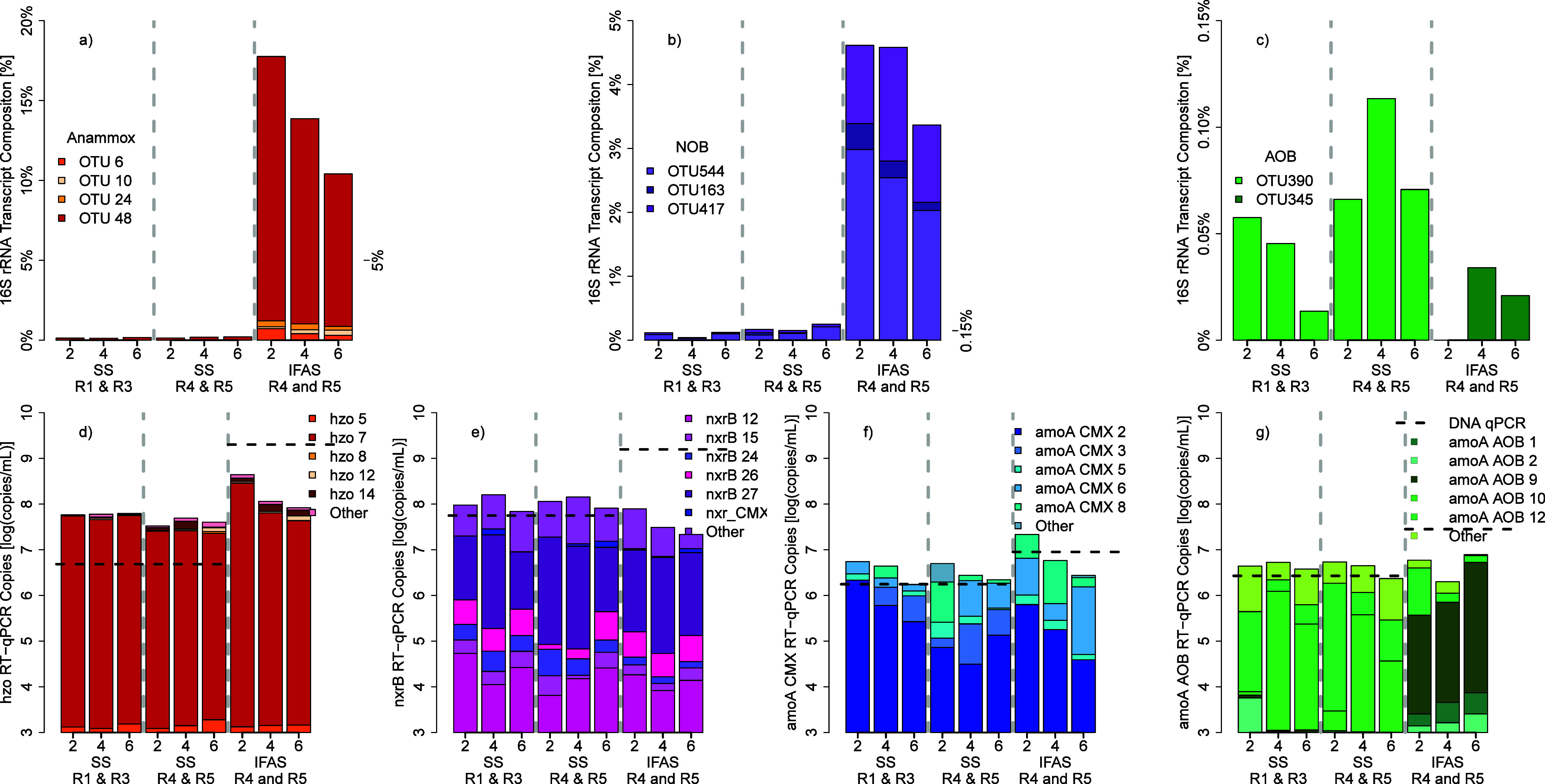
Summary of dominant nitrifying bacteria’s transcriptional activity based on 16S rRNA transcript sequencing (a,b,c) and functional gene expression (d–g). 16S rRNA transcript activity with (a) anammox bacteria, (b) all nitrite oxidizing bacteria (including comammox which cannot be differentiated using the OTUs of 16S rRNA V4 amplicons), and (c) strict ammonia oxidizing bacteria. The expression of functional genes are quantified via RT-qPCR and then multiplied with OTU relative abundance in amplicon sequencing data (*y*-axis) for (d) hydrazine oxidoreductase found in AMX, (e) nitrite oxidoreducatase of all nitrite oxidizing bacteria (including comammox *Nitrospira*), (f) ammonia monooxygenase found in comammox *Nitrospira*, and (g) ammonia monooxygenase found in strict ammonia oxidizing bacteria. The dashed line represents the average abundance of each functional gene via qPCR to determine transcripts-to-gene ratios.

The diversity of nitrifying bacteria was analyzed by using a combination of 16S rRNA transcript sequencing and quantification and amplicon sequencing of major nitrogen-cycling functional genes (Figure 2). 16S rRNA transcript analysis averaged 63,049 ± 11,365 postprocessed reads per sample (Figure S3) and revealed one dominant AMX (Figure 2a), two dominant AOB (Figure 2c), and three *Nitrospira*-like OTUs (Figure 2b).

Anammox activity was dominated by that of OTU48, *Candidatus* Brocadia, based on 16S rRNA transcript quantification. OTU48 was particularly active in IFAS and comprised 16.5% ± 4.2% of the 16S rRNA transcript composition at low DO and declined down to 9.5% ± 2.2% at higher DO concentrations. Similarly, there was a single OTU, *hzo* 7, which dominated anammox activity based on *hzo* transcript absolute abundance (Figure 2d) and consistently comprised 93.43% ± 2.85% of the *hzo* transcript composition. IFAS had significantly higher *hzo* expression at low DO (ANOVA *p* = 0.00007, *R*^2^ = 0.79) at 8.64 ± 0.13 log(copies/mL) and decreased with higher DO to 7.92 ± 0.13 log(copies/mL). AMX’s *hzo* expression was stable in the suspended sludge biomass at 7.69 ± 0.24 log(copies/mL) and not impacted by DO concentration (ANOVA *p* = 0.337). While AMX preferring lower DO concentrations has been previously reported,^24,64^ what is interesting when comparing our system is the lack of diversity in AMX, whereas typically multiple species of abundant AMX^28^ are observed to co-occur from wastewater bioreactors seeing Ca. *Brocadia* and Ca. *Kuenenia* together,^64^ to rice-wheat paddy fields observing Ca. *Brocadia* and Ca. *Jettenia*.^65^ While there are low abundance species observed (Figure S4), these species are also orders of magnitude lower in abundance and lower levels of activity.

Three dominant *Nitrospira* OTUs were observed based on 16S rRNA transcript analysis: OTU544 closely associated with *Nitrospira* defluvii, OTU163 associated with *Nitrospira moscoviensis*, and OTU417 with an unknown *Nitrospira* (Figure 2b). For redundancy, 16S rRNA sequences were compared across SILVA SSU 138.1 and MIDAS 5.0 databases to assess whether CMX-associated 16S rRNA sequences were distinguishable from NOB, but this was not feasible (data not shown). All 16S rRNA transcripts for *Nitrospira* were significantly more active in the IFAS biofilm than in suspended sludge biomass (ANOVA_defluvii_*p* = 0.0001, *R*^2^ = 0.77, ANOVA_moscoviensis_*p* = 0.000005, *R*^2^ = 0.87, ANOVA_na_*p* = 0.0049, *R*^2^ = 0.26).

In contrast to the 16S rRNA transcript data, which showed remarkable differences in *Nitrospira* (both CMX and NOB) activity in IFAS biofilm as compared to suspended sludge biomass, RT-qPCR data indicates relative stable transcriptional activity of the *nxrB* gene in the suspended sludge biomass (7.99 ± 0.23 log(copies/mL)) which was comparable to that of the IFAS biofilm-based *nxrB* activity (Figure 2e). Interestingly, *nxrB* transcript abundance dropped significantly with increasing dissolved oxygen (ANOVA *p* = 0.0002, R^2^ = 0.83) from 7.89 ± 0.08 to 7.31 ± 0.12 log(copies/mL) from DO = 2 mg/L up to DO = 6 mg/L in the IFAS biofilm community. If *nxrB* activity was only associated with nitrite oxidation, this drop would be perplexing, since ammonia oxidation is higher at high dissolved oxygen concentrations. However, this drop in *nxrB* transcripts could be indicative of lower nitrite concentrations found in IFAS biofilm since the available nitrite is likely consumed by AMX or alternatively in anoxic or low DO conditions *nxrB* could reversibly reduce nitrate back to nitrite. This would explain the higher *nxrB* transcripts in the IFAS biofilm at lower dissolved oxygen concentrations. Similarly to 16S rRNA amplicon sequencing, it was not possible to distinguish CMX *Nitrospira nxrB* amplicons from strict NOB *Nitrospira,* necessitating `metatranscriptomic evaluation.

To determine if any *nxrB* transcripts were from CMX, the *nxrB* transcripts were mapped to all *Nitrospira* MAGS. Several low abundance *nxrB* OTUs mapped to CMX accounted for 1.02% ± 0.84% of the *nxrB* transcript composition without significant changes due to DO concentration (ANOVA *p* = 0.83). Interestingly, the *amoA* CMX activity was significantly higher in the IFAS biofilm compared to the suspended phase and demonstrated sensitivity to DO concentration (ANOVA *p* = 0.033) increasing from 6.43 ± 0.32 log(copies/mL) up to 7.33 ± 0.58 log(copies/mL) with a drop in DO from 6 to 2 mg/L (Figure 2f). Overall, in suspended sludge biomass, CMX appears to have a stable transcriptional activity of *nxrB* and a low activity of *amoA*, whereas in IFAS biofilm, a significant increase in *amoA* activity is observed. Interestingly, the CMX *amoA* activity did significantly increase at reduced DO, even in suspended sludge biomass. This is uncommon since *amoA* activity is regulated by ammonia concentration, not oxygen concentration.^66^ This change in expression in the suspended phase is likely due to residual *amoA* transcripts of biomass being sloughed off from IFAS biofilm into the suspended phase which would experience fluctuations in ammonia concentration, therefore impacting *amoA* transcript regulation based on DO concentration. This aligns with current studies showing that CMX strongly prefers biofilms^67^ and our previous work using intrinsic assays and differential inhibition to suggest higher ammonia oxidation from CMX in IFAS biofilm.^4^ Other studies using differential inhibitors with CMX enrichment cultures from rotating biological contactors have shown similar changes in CMX activity between biofilm and suspended sludge biomass cultures.^68^ While our study suggests higher nitrite oxidation activity of CMX in suspended sludge biomass based on stable and higher expression of *nxr* genes relative to that of the attached phase, future studies are required to investigate the mechanisms underpinning CMX differential expression in suspended and attached phases. Further, it is also important to note that the residual ammonia concentration in the aerated zone increased with a drop in DO concentration due to incomplete nitrification as reported previously.^4^ We are unable to clearly discriminate between these covariates (i.e., change in nitrogen species concentration associated with DO concentration set point), as part of this full-scale study.

Two dominant strict AOB (OTU390, OTU345) within the family *Nitrosomonadaceae* were detected in the 16S rRNA transcript amplicon sequencing library. While both OTUs showed very low levels of activity based on their relative abundance in the 16S rRNA transcript libraries, OTU390 was active only in the suspended phase, while only OTU345 demonstrated low levels of activity in the IFAS biofilms (Figure 2c). While the absolute abundance of AOB was significantly higher in IFAS biofilm samples (7.45 ± 0.9 log(copies/mL)) compared to suspended sludge biomass samples (6.42 ± 0.26 log(copies/mL)), their transcriptional activity based on the RT-qPCR assay targeting the *amoA* gene indicated no significant change with either reactor zonation or DO concentration (ANOVA_DO_ = 0.426, ANOVA_zone_ = 0.839) averaging at 6.63 ± 0.31 log(copies/mL) (Figure 2g). This indicates a significant downregulation of *amoA* transcriptional activity (on a per cell basis) in the IFAS biofilm community. This stable activity of *amoA* by AOB is consistent with past activated sludge systems^69−71^ but overall lower than typically reported between 7 and 8 log(copies/mL). This is consistent with our previous findings that CMX and AMX significantly contribute to nitrogen removal, especially in IFAS biofilm phase.^4^

Multiple AOB-related *amoA* OTUs were identified in both the suspended sludge biomass and the IFAS biofilm phase. This contrasts with the two 16S rRNA gene AOB OTUs, where only one OTU was observed in each of phase. This could likely emerge due to both the presence of multiple *amoA* gene copies within genomes relative to a single 16S rRNA gene per AOB genome^72^ but also could emerge from the differences in the sensitivities of two assays. Specifically, *amoA* gene amplicon sequencing was performed after selectively amplifying this gene from cDNA, and as a result, it is likely to detect sequences from more rare populations as compared to when the entire microbial community is profiled using 16S rRNA gene assay. Nonetheless, in alignment with the 16S rRNA transcript data, a single OTU, *amoA 9,* was primarily active in the IFAS biofilm community. This *amoA 9* transcript exhibited sensitivity to DO concentration increasing from 57.2% at 2 mg/L to 74.4% of all AOB *amoA* transcripts at 6 mg/L. Furthermore, the *amoA* 9 OTU sequence exhibited 99.6% sequence similarity with an uncultured *Nitrosomonas clone A12*.^73^ This *Nitrosomonas clone A12* was previously found in a biofilm working alongside CMX and raises the possibility that specific *Nitrosomonas* species are more inclined to coexist with CMX or potentially both prefer biofilms due to their growth kinetics.

### 3.3 Comammox Nitrospira and Anammox Bacteria Have Strong Synergistic Relationship in the IFAS Biofilm Phase

**Figure 3:**
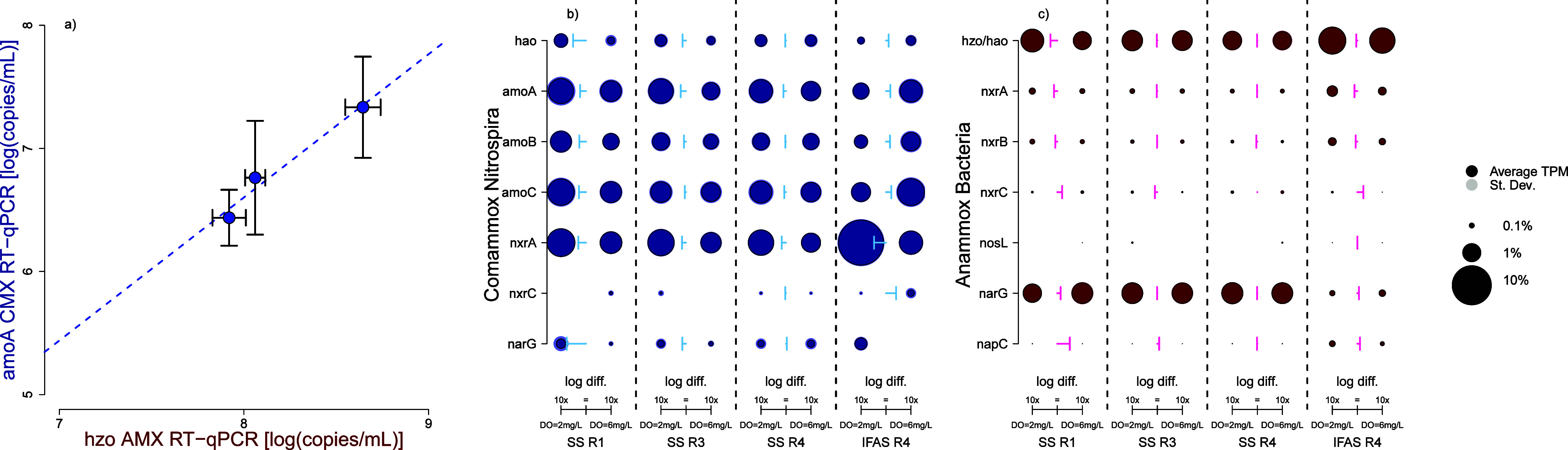
Anammox bacteria and comammox *Nitrospira* have a strong linear relationship of their activity in IFAS biofilm based on (a) RT-qPCR transcript quantification of hydrazine oxidoreductase and ammonia monooxygenase from comammox Nitrospira. This symbiotic relationship was further explored using transcripts-per-million activity profiles of major nitrogen cycling pathways mapped to MAGS constructed of (b) comammox *Nitrospira* and (c) anammox bacteria. The size of each point relates to the percent of transcripts per million, with a shaded background for standard deviation. The arrows show the magnitude difference between each data point DO = 2 mg/L and DO= 6 mg/L.

CMX and AMX show a strong symbiotic relationship in IFAS biofilm as indicated by significant correlative relationships (Figure 3a) and transcripts-per-million gene expression analysis of major nitrogen cycling pathways (Figure 3b,c). Using RT-qPCR transcript abundances, *hzo* transcripts were regressed against *amoA* for CMX transcripts in IFAS biofilm (Figure 3a) and a significant correlation was observed (Spearman correlation *R* = 0.69, *p* = 0.03), suggesting that AMX *hzo* and CMX *amoA* activities may be potentially coregulated by changes in DO concentrations. While similar regressions can be performed using *nxrB,* CMX *amoA* and AMX *hzo* are the only genes that exhibited significantly higher transcriptional activity in the IFAS biofilm relative to that in the suspended phase. This contrasts with expression levels of AOBs *amoA* and total *nxrB* transcripts, which were either stable or declined in the IFAS biofilm, respectively. This suggests that aerobic ammonia oxidation by CMX was strongly associated with anaerobic ammonia oxidation by AMX, suggesting that CMX may play a role in supplying nitrite to AMX. This has been previously suggested since the reported discovery of CMX coexisting with an AMX population,^74,75^ but this study is the first to demonstrate this symbiotic relationship leveraging transcriptomic expression at full-scale. This is exceptionally notable since other treatment plants have been inoculated with anammox pellets while having multiple types of nitrifying bacteria including comammox *Nitrospira* as the dominant nitrifying bacteria only for anammox to be suppressed and undetected over time.^32^

This relationship was further investigated by examining differential expression of key nitrogen cycling genes in MAGS from CMX (Figure 3b) and AMX (Figure 3c) by mapping meta-transcriptomic reads to them. Similar analyses for AOB and NOB can be found in the Supporting Information (Figure S6). Nitrogen cycling genes from CMX were most impacted by changes in DO concentration with significant increases in *nxrA* across every reactor setting (Tukey_SSR1_DO2-SSR1_DO6_*p* = 4.57 × 10^–2^, Tukey_SSR3_DO2-SSR3-DO6_*p* = 6.29 × 10^–3^, Tukey_SSR4_DO2-SSR4_DO6_*p* = 6.93 × 10^–3^, Tukey_IFAS_DO2-IFAS_DO6_*p* = 2.82 × 10^–14^) at lower DO concentrations. In suspended sludge biomass, nitrogen cycling pathways accounted for 7.75% ± 1.62% of the CMX transcripts at low DO concentrations and only 4.31% ± 0.85% at high DO concentrations. This was further highlighted when comparing activity in IFAS biofilm with 18.53% ± 2.89% and 7.18% ± 1.40% at DO = 2 mg/L and DO = 6 mg/L of all transcripts, respectively, mapped to nitrogen cycling pathways. The largest increase in activity was associated with *nxrA* in IFAS biofilm, which increased 9.1-fold at low DO concentrations. This was surprising since *nxrC* expression did not significantly change between DO concentrations and was often undetected, but this may be a result of a fragmented MAG resulting in low mapped transcripts since other studies have shown consistent transcriptional activity across all *nxr* subunits.^76−78^ Further work needs to investigate biofilm depth-dependent activities and oxygen gradients to understand how CMX regulates the expression of nitrogen cycling genes across a redox gradient.^79^

AMX maintained mostly consistent expression of nitrogen cycling genes in suspended sludge biomass comprising 2.52% ± 0.19% of the total mapped AMX transcripts (Figure 3c). In comparison to the IFAS biofilm, *narG* (nitrate reductase) was significantly more expressed in suspended sludge biomass (Tukey *p* = 1.79 × 10^–14^) as compared to the IFAS biofilm. This suggests that in suspended sludge biomass AMX may need to supply their own nitrite from residual nitrate due to competition for nitrite with NOB. The downregulation of *narG* in the IFAS biofilm could indicate a sufficient supply of nitrite, likely from CMX. Additionally, in the IFAS biofilm, most pathways experienced a significant increase in transcripts-per-million at low DO concentrations. The expression of genes associated with the *hao*/*hzo* pathway increased by 1.2-fold, *nxrA* increased by 1.68-fold, and *nxrB* increased by 1.36-fold. These increases resulted in AMX’s nitrogen cycling transcripts in IFAS biofilm increasing from 2.65% ± 0.23% at DO = 6 mg/L up to 3.23% ± 0.08% at DO = 2 mg/L (Tukey_IFAS-SS_*p* = 5.03 × 10^–13^, Tukey_IFAS_DO2-IFAS_DO6_*p* = 1.59 × 10^–9^, Tukey_ss_DO2-SS_DO6_*p* = 5.91 × 10^–1^) with overall activity in IFAS biofilm being significantly higher than in suspended sludge biomass.

### 3.4 Differential Gene Expression Analysis Indicates that AMX Increases Flagellar Activity in IFAS Biofilm Phase under High DO Conditions

**Figure 4:**
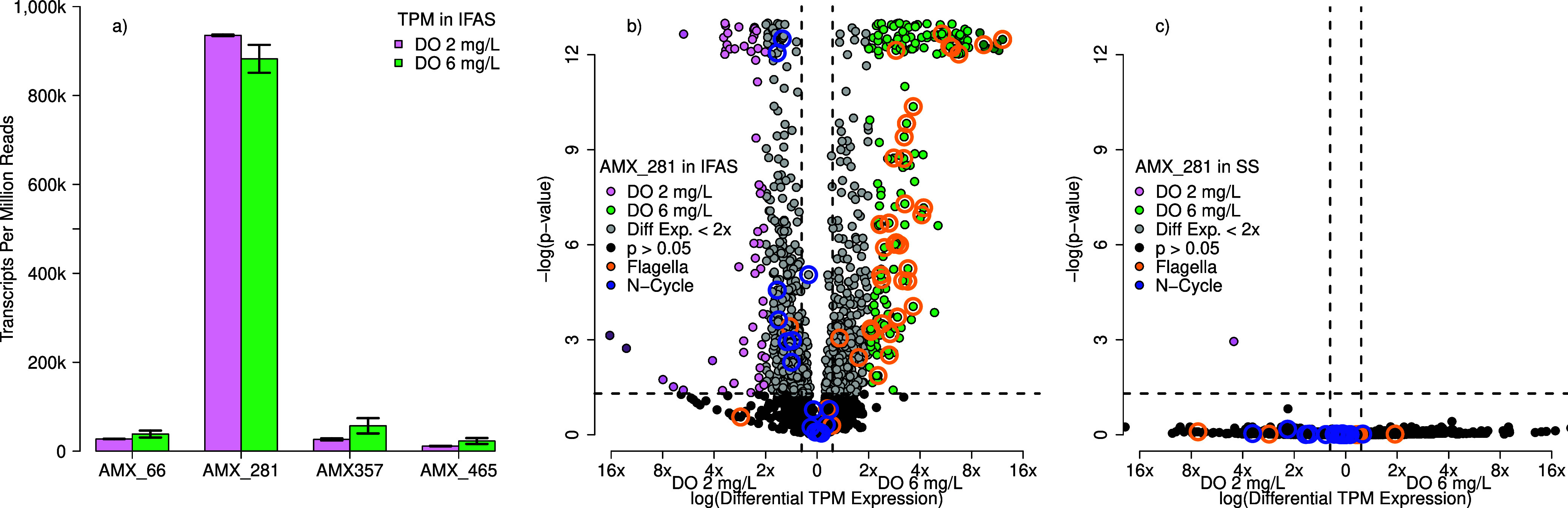
Anammox MAG, AMX_281, was highly active in IFAS biofilm compared to other MAGS (a), and further analyzed using DESeq2 for differential gene expression analysis in (b) IFAS biofilm and (c) suspended sludge biomass. The reported *p*-values were adjusted by using the Benjamini–Hochberg multiple comparison correction. Points in pink were statistically significantly more expressed at DO = 2 mg/L while points in green were statistically significantly more expressed at DO = 6 mg/L. Blue circles represent shifts in nitrate transporting, while the orange circles represent transcripts related to flagella activity. In instances where the -log(p-value) exceeded 12, they are plotted as 12.5 with a jitter for spacing.

AMX_281 was the most actively transcribed MAG in IFAS biofilm with 93% ± 0.2% of the transcripts mapping at DO = 2 mg/L, and 88.3% ± 3.1% at DO = 6 mg/L (Figure 4a). This is consistent with dominance of 16S rRNA transcript OTU 48 whose transcripts completely mapped to the MAG, and the dominance of *hzo* transcript OTU 7, which did not map to the MAG due to the *hzo* gene within the genome being a partial gene without a sequence overlap with the *hzo* amplicons.

AMX_281 was further analyzed in IFAS biofilm (Figure 4b) and in suspended sludge biomass (Figure 4c) with genes considered significantly differentially transcribed with a Wald test *p* < 0.05 and greater than 2× transcripts per million between two conditions being compared (i.e., 2 vs 6 mg/L of DO or biofilm vs suspended phase). Most genes associated with nitrogen cycling functions in AMX_281 showed insignificant changes in relative expression abundance, similar to all AMX in Figure 3c. If nitrogen cycling genes are up-regulated as shown with *hzo* quantification via RT-qPCR in Figure 2d, they increase proportionally to maintain similar relative expression via transcripts per million and differential gene expression (Figures 3c and 4b). At lower DO concentrations, most up-regulated gene expression was from hypothetical protein-coded sequences and was not further investigated.

At high dissolved oxygen concentrations in the IFAS biofilm, there was a significant increase in most flagella-related transcriptional activity, which was not similarly observed in suspended sludge biomass. This was observed for genes across multiple flagellar-related proteins from FlgN, FliS, Flagellar hook-associated protein 2, and FlaG, all of which increased more than 5-fold at high DO concentrations. Yan et al.^24^ suggested that this may be an oxygen-related stress response and trigger biofilm formation. While reactive oxygen species could impact AMX in IFAS biofilm, this stress response is not consistent with AMX in suspended sludge biomass (Figure 4c), which did not show significant changes in flagella-related activities and accounted for an overall lower abundance of transcripts-per-million (IFAS_DO6_ = 1.78%, SS_DO6_ = 0.08%). We hypothesize that anammox bacteria in IFAS biofilms may increase mobility due to chemotaxis and cell growth within the competitive environment of a biofilm. Research into anammox located in deep-sea anoxic nitrate-ammonium transition zones observed expression of full gene sets for flagella mobility despite limited energy for mobility, suggesting flagellar activity is linked to *in situ* growth.^80^ Similarly, other *Planctomycetes* bacteria have shown increased flagella mobility in highly rich media,^81^ suggesting that AMX may utilize chemotaxis within high nutrient concentrations as opposed to mobility to avoid oxygen stress.^82^

### 3.5 Comammox Nitrospira Alters Ammonia and Nitrite Metabolic Preferences Based on Redox Conditions

**Figure 5:**
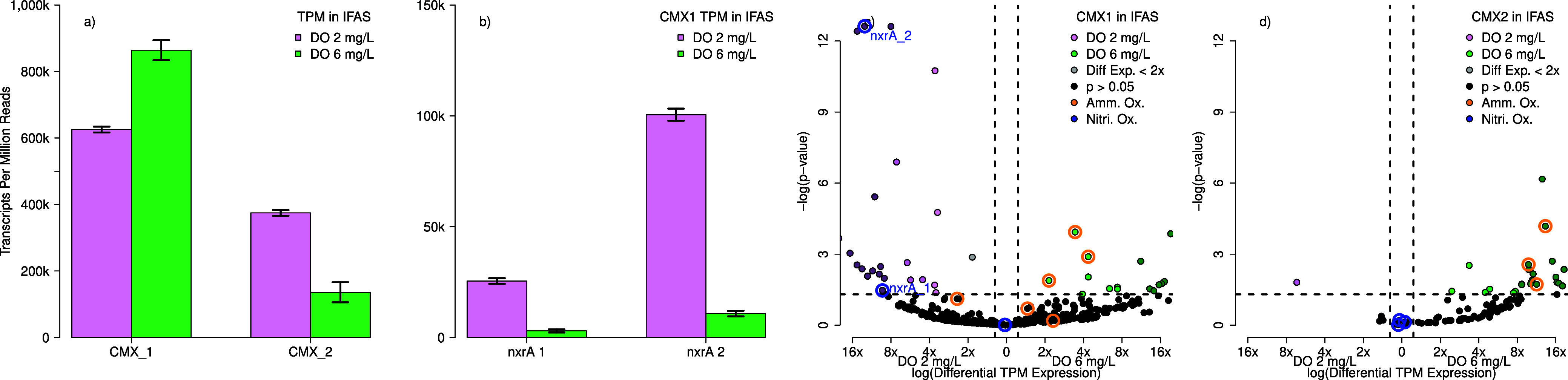
Comammox Nitrospira had two dominant MAGS in IFAS biofilm (a), which were further analyzed using volcano plots for differential gene expression analysis in the IFAS biofilm. The more abundant MAG, CMX_1, saw significant changes in the expression between the two copies of *nxrA* within the genome (b). Each MAG was analyzed with DESeq2 for differential gene expression analysis in IFAS biofilm with (c) CMX_1 and (d) CMX_2. The reported *p*-values were adjusted using the Benjamini–Hochberg multiple comparison correction. Points in pink were statistically significantly more expressed at DO = 2 mg/L while points in green were statistically significantly more expressed at DO = 6 mg/L. Blue circles represent shifts in nitrite oxidation activity, while the orange circles represent shifts in ammonia oxidation activity. In instances where the −log(*p*-value) exceeded 12, they are plotted as 12.5 with a jitter for spacing.

Two CMX MAGS revealed a significant shift in metabolic preferences based on DO concentration (Figure 5). Transcripts-per-million analysis was performed to compare the activity of CMX_1 and CMX_2. CMX_1 was overall more active than CMX_2 comprising 62.5% ± 0.9% of transcripts per million at DO = 2 mg/L and 86.4% ± 3.0% at DO = 6 mg/L, whereas CMX_2 comprised 37.5% ± 0.9% at DO = 2 mg/L and 13.6% ± 3% at DO = 6 mg/L, respectively (Figure 5a). Further, genes associated with ammonia oxidation were significantly upregulated at 6 mg/L DO, while genes associated with nitrite oxidation were significantly upregulated at 2 mg/L DO.

The CMX_1 MAG (Figure 5c) showed significant increases in transcription of genes involved in nitrite oxidation pathways at lower DO concentrations in the IFAS biofilm, further reinforcing *nxrB* increases shown in Figure 3b. The relative downregulation of *amoA* in CMX gene expression conflicts with *amoA* transcript quantification in CMX via RT-qPCR (Figure 2f), which showed an increase in expression. This apparent difference in the expression of *amoA* and *nxrB* in CMX is a result of comparing a relative expression metric (transcripts-per-million) to an absolute expression metric (RT-qPCR). At low DO levels, CMX increases the expression of genes involved with both ammonia oxidation and nitrite oxidation, but those associated with nitrite oxidation have a much larger increase, resulting in the expression of genes associated with ammonia oxidation appearing to decrease using relative expression metrics.

CMX_1 had sufficient mapped transcripts to compare differential expressions between the two copies of *nxrA* within the genome. The second copy of *nxrA* had significantly more actively expressed than the first copy of *nxrA* (Tukey p = 5.11e-6). Furthermore, the ratio of expression between the second copy and the first copy was constant at 4.14, and there were no significant differences in the ratio based on DO concentration (Tukey *p* = 0.739).—Different regulations for the paralogs have been previously observed in cultures^83^ but not previously reported in a full-scale wastewater treatment system.

Interestingly, the NXR enzyme is also a reversible, which can either oxidize nitrite or reduce nitrate in anaerobic conditions.^84^ This dynamic further reinforces the synergistic relationship shown in Figure 3 where CMX are likely supplying AMX with nitrite in IFAS biofilm as previously reported by Vilardi et al.^4^ In aerobic conditions, CMX could perform ammonia oxidation and supply nitrite to AMX. In anaerobic conditions, the NXR enzyme could be reversed for nitrate reduction to resupply nitrite to AMX. Hydrogen gas could be a potential electron donor since comammox *Nitrospira* have been shown to have hydrogenases,^31^ which were also present in CMX_1, and low DO conditions may result in some level of fermentative activity. Unfortunately, our limited RNA-seq depth was unable to show the transcriptomic activity for hydrogenase pathways. These CMX MAGs^4^ did contain multiple subunits of [NiFe] hydrogenase.^85^ Future research is required to distinguish if nitrate reduction can be carried out by comammox *Nitrospira.*

These scenarios could explain why CMX significantly increases ammonia oxidation and greatly decreases *nxr* expression in IFAS biofilm, whereas strict NOB do not significantly increase *nxrA* expression in IFAS biofilm phase (Figure S6c). Furthermore, there is an overall decrease in *nxrB* expression in IFAS biofilm compared to suspended sludge biomass (Figure 2e) since CMX dominates the *Nitrospira* community in IFAS biofilm and strict NOB dominate the suspended sludge biomass. While others have reported differential expression of *amo* and *nxr* in CMX,^76,77^ this would be the first report suggesting the role of DO and redox zonation promoting differential expression.

### 3.6 Implications for Development of a CMX-AMX System for Mainstream Nitrogen Removal and Future Research

The cooperation of CMX and AMX in IFAS biofilms at lower DO concentrations has significant beneficial implications for wastewater treatment facilities. Such coupling effectively doubles as both cost saving due to lower aeration energy consumption^27^ and decarbonization by potentially minimizing the release of nitrous oxide during nitrification.^6^ Further, integrating an IFAS biofilm component within a conventional nitrification-denitrification process does not require significant infrastructure changes and can be accomplished by integrating carrier media within existing reactors. The metabolic flexibility of CMX serves three critical roles benefiting AMX by (1) aerobically converting ammonia to nitrite for AMX’s anaerobic ammonia oxidation, (2) potentially anaerobically converting nitrate back to nitrite for further AMX anaerobic ammonia oxidation, and (3) utilizing oxygen via aerobic ammonia oxidation and nitrite oxidation to create anaerobic zones for AMX. While this study demonstrates that nitrifying activities of CMX and their cooperation with AMX are sensitive to DO concentrations, it also opens up further research questions and needs for process optimization. What is the optimal DO concentration to maximize anaerobic ammonia removal considering the more pronounced upregulation of genes associated with nitrite oxidation compared to ammonia oxidation for CMX bacteria at lower DO concentrations? What role does partial denitrification play in nitrite provision for AMX under low DO conditions? Further, the conditions critical for the establishment and maintenance of AMX and CMX cooperation in a full-scale mainstream system are as yet unknown since our work was conducted on already established nitrifying community.

## Data Availability

All code for amplicon sequencing, metatranscriptomic analysis, statistical analysis and plots are available at github.com/queermsfrizzle/CMX_AMX_transcriptomics. Raw fastq files for metatranscriptomic sequencing and amplicon sequencing are available via NCBI bioproject submission number PRJNA1050761.

## Supporting Information Available

Table of primers for qPCR, RT-qPCR and amplicon sequencing, table of top blast query for amplicons, figure of RT-qPCR gene expression, figure of normalization by copies per volume and copies per mass, figure of filtered reads frequency, figure of Shannon diversity index, figure of amplicon composition by OTUs, figure of all nitrifying bacteria’s transcript per million for nitrification pathways, and text focused on strict ammonia-oxidizing bacteria and strict nitrite-oxidizing bacteria in suspended sludge (PDF)
