# Supplementary material for: Metatranscriptomic Analysis Reveals Synergistic Activities of Comammox and Anammox Bacteria in Full-Scale Attached Growth Nitrogen Removal System
Source: Environ Sci Technol. 2024 Jul 13;58(29):13023–34. doi: 10.1021/acs.est.4c04375 (PMC11271001; doi:10.1021/acs.est.4c04375)
Supplement: Supplementary file 1 — es4c04375_si_001.pdf [file es4c04375_si_001.pdf]

SUPPORTING INFORMATION

Metatranscriptomic analysis reveals synergistic activities of comammox and anammox bacteria in full-scale attached growth nitrogen removal system

Authors: Juliet Johnston[1], Katherine Vilardi[2], Irmarie Cotto[3], Ashwin Sudarshan[1], Kaiqin Bian[1], Stephanie Klaus[4], Megan Bachmann[4,5], Mike Parsons[4], Christopher Wilson[4], Charles Bott[4], Ameet Pinto[1*]

[1]School of Civil and Environmental Engineering, Georgia Institute of Technology, Atlanta, Georgia, 30332, USA

[2]Department of Civil and Environmental Engineering, Northeastern University, Boston, Massachusetts, 02115, USA

[3]Department of Environmental and Occupational Health Sciences, University of Washington, Seattle, Washington, 98195, USA

[4]Hampton Roads Sanitation District, Virginia Beach, Virginia, 23455, USA

[5]Department of Civil and Environmental Engineering, Virginia Tech, Blacksburg, Virginia, 24061, USA

*Corresponding author: Ameet Pinto (ameet.pinto@ce.gatech.edu)

This supporting information is 11 pages with two tables, six figures, and one page of supplemental text.

Table S1: Primer sequences and amplicon lengths used for qPCR, RT-qPCR, and amplicon gene sequencing analysis.

Target	Name	Length [bp]	Forward [5'-3']	Reverse [5'-3']	Citation
16S rRNA V4	515F / 806R	292	GTGYCAGCMGCC GCGGTAA	GGACTACNVGGG TWTCTAAT	Walters 2015[25]
amoA AOB	amoA-1Fmod / amoA_2R	491	CTGGGGTTTCTAC TGGTGGTC	CCCCTCKGSAAA GCCTTCTTC	Meinhardt 2015[26]
amoA CMX	496F / 812R	345	GCGATTCTGTTTT ATCCCAGCAAC	CCGTGTGCTAAC GTGGCG	Beach 2019[27]
nxrB	nxrB169f / nxrB638r	485	TACATGTGGTGGA ACA	CGGTTCTGGTCR ATC	Pester 2013[28]
hzo	hzocl1F1 / hzocl1F2	471	TGYAAGACYTGY CAYTGG	ACTCCAGATRTG CTGACC	Yang 2020[29]

Table S2: The top BLAST query, sorted based on lowest E-value for representative sequences from each OTU cluster. The matching sequence ID listed with the percent identity similarity.

OTU	Blast Description	Sequence ID	Percent Identity
**amoA AOB 1**	Uncultured Clone BB405	KJ562489.1	99.39%
**amoA AOB 2**	Uncultured Clone QH2 55	KT023767.1	99.59%
**amoA AOB 9**	Nitrosomonas sp. Clone A12_beta	KX024803.1	99.39%
**amoA AOB 10**	Uncultured Clone amoA_SBR_JJY_11	FJ577851.1	99.80%
**amoA AOB 12**	Nitrosococcus sp. Clone SBA21	KC769050.1	99.59%
**amoA CMX 2**	Uncultured Nitrospira sp. Clone ZSCMX	ON551360.1	98.84%
**amoA CMX 3**	Uncultured Nitrospira sp. Clone ZSCMX	ON551360.1	98.26%
**amoA CMX 5**	Uncultured Nitrospira sp. Clone ZSCMX	ON551360.1	99.71%
**amoA CMX 6**	Uncultured Nitrospira isolate RBC Group J	LR723642.1	98.55%
**amoA CMX 8**	Uncultured Nitrospira isolate RBC Group J	LR723642.1	97.39%
**nxrB NOB 12**	Uncultured Nitrospira sp. Clone HKA-E8	KC884878.1	99.58%
**nxrB NOB 15**	Uncultured bacterium nsxB-31	AB846874.1	99.58%
**nxrB NOB 24**	MAG: Nitrospira sp. Isolate UBC3 chromosome	CP092079.1	97.32%
**nxrB NOB 26**	Uncultured bacterium nsxB-04	AB846849.1	99.17%
**nxrB NOB 27**	Uncultured bacterium NOB OTU149 NLJ4_2973	MW343074.1	98.68%
**nxrB CMX 17**	Uncultured prokaryote nxrB	LC257062.1	98.01%
**hzo AMX 5**	Uncultured planctomycete ANAHZO-4	EU294367.1	98.94%
**hzo AMX 7**	MAG: Planctomycetia isolate H1_AMX1	CP054188.1	99.57%
**hzo AMX 8**	MAG: Planctomycetia isolate H1_AMX1	CP054188.1	95.10%
**hzo AMX 12**	Candidatus Brocadia pituitae complete genome	AP021856.1	99.15%
**hzo AMX 14**	MAG: Brocadia sp. Ega_18_Q3-R5-49_MAXAC.112v2	CP064969.1	98.72%

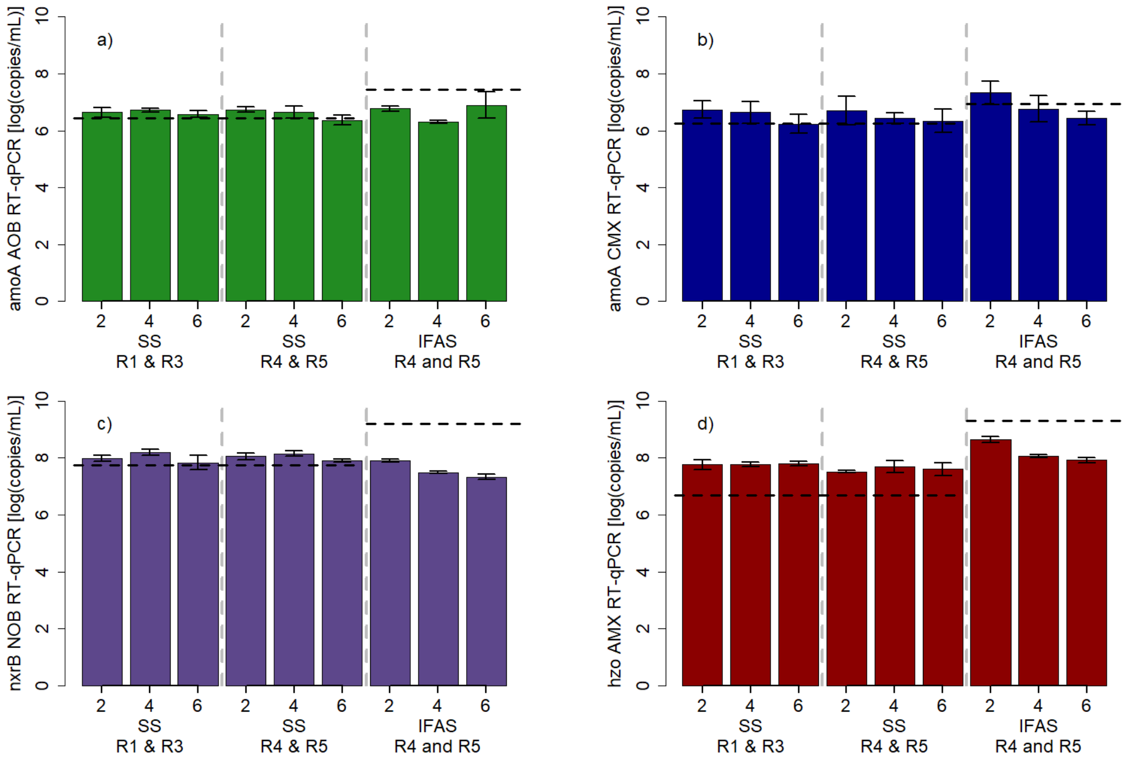

Figure S1: Quantification of functional gene expression via RT-qPCR for a) *amoA* in strict ammonia oxidizing bacteria b) *amoA* in Commamox Nitrospira c) *nxrB* in all nitritie oxidizing bacteria, and d) *hzo* in anammox bacteria. SS = suspended sludge, IFAS = IFAS biofilms. The standard deviations are shown with the error bars and the average functional genes abundance via qPCR is shown with the dashed lines.

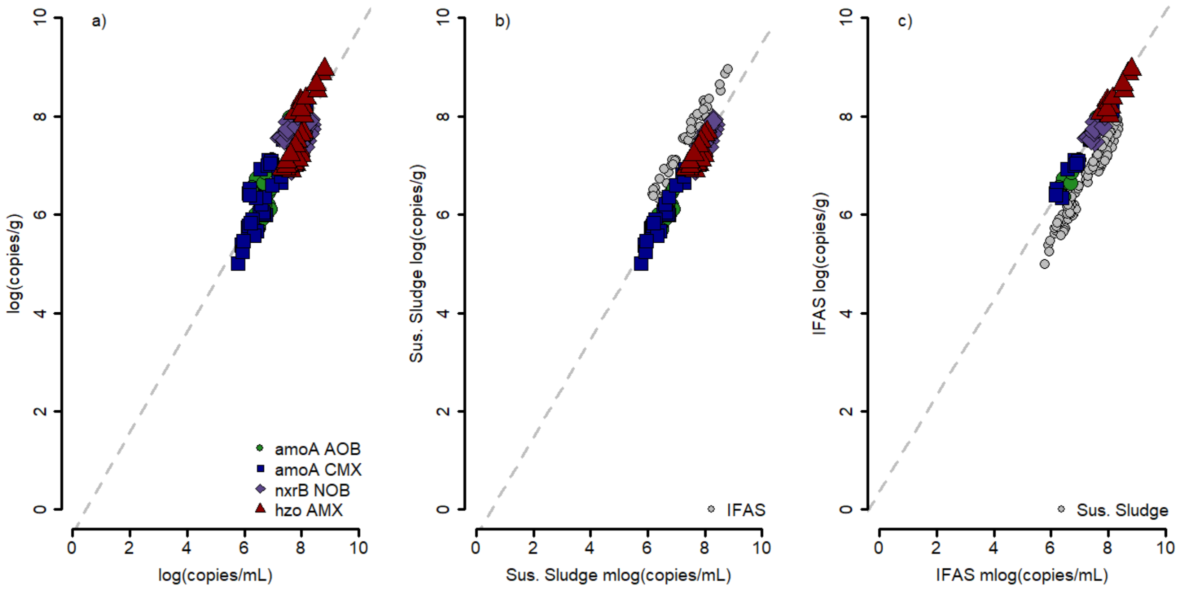

Figure S2: Comparison of normalization techniques between using copies per volume vs copies per mass of sludge. Suspended sludge b) has a strong linear relationship with $R^2 = 0.963$ and $p << 0.05$. The average ratio of log(copies/gram)/log(copies/mL) was 0.927 ± 0.022 for suspended sludge. In IFAS c), the strong linear relationship was also observed with an $R^2 = 0.972$ and $p << 0.05$. The average ratio of log(copies/gram)/log(copies/mL) was higher 1.024 ± 0.018 because the biomass on IFAS was significantly more dense than the suspended sludge.

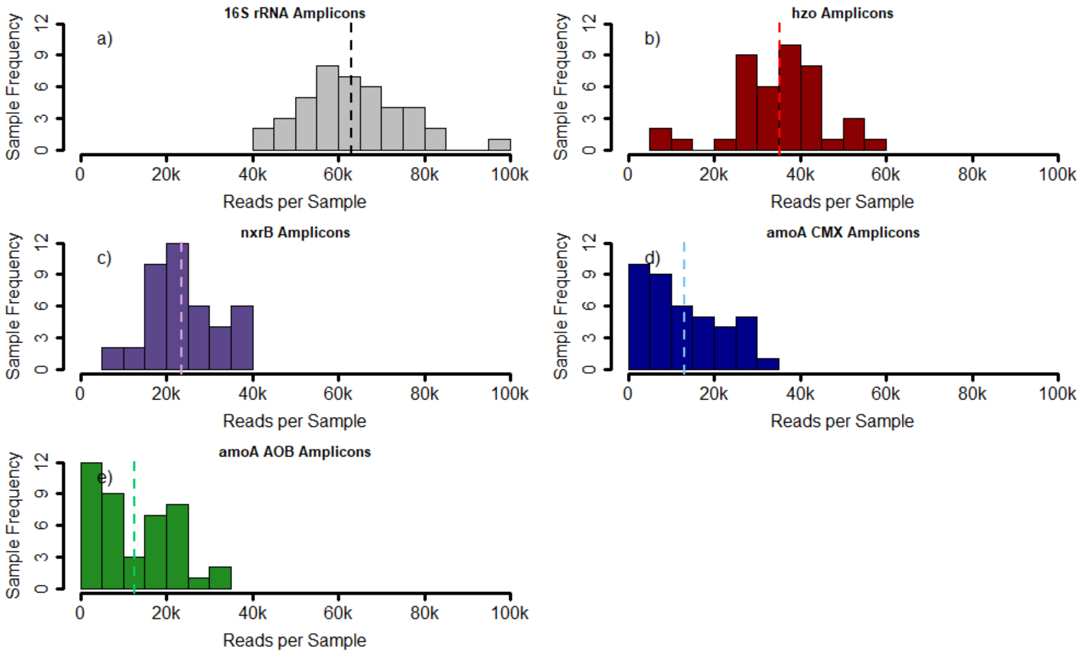

Figure S3: The frequency and distribution of post-processed and filtered reads a) 16S rRNA transcript sequences, b) *hzo* transcript sequences, c) *nxrB* transcript sequences, d) *amoA* for Comammox *Nitrospira* transcript sequences and e) *amoA* for strict ammonia oxidizing bacteria transcript sequences. Dashed lines indicate the average reads per sample.

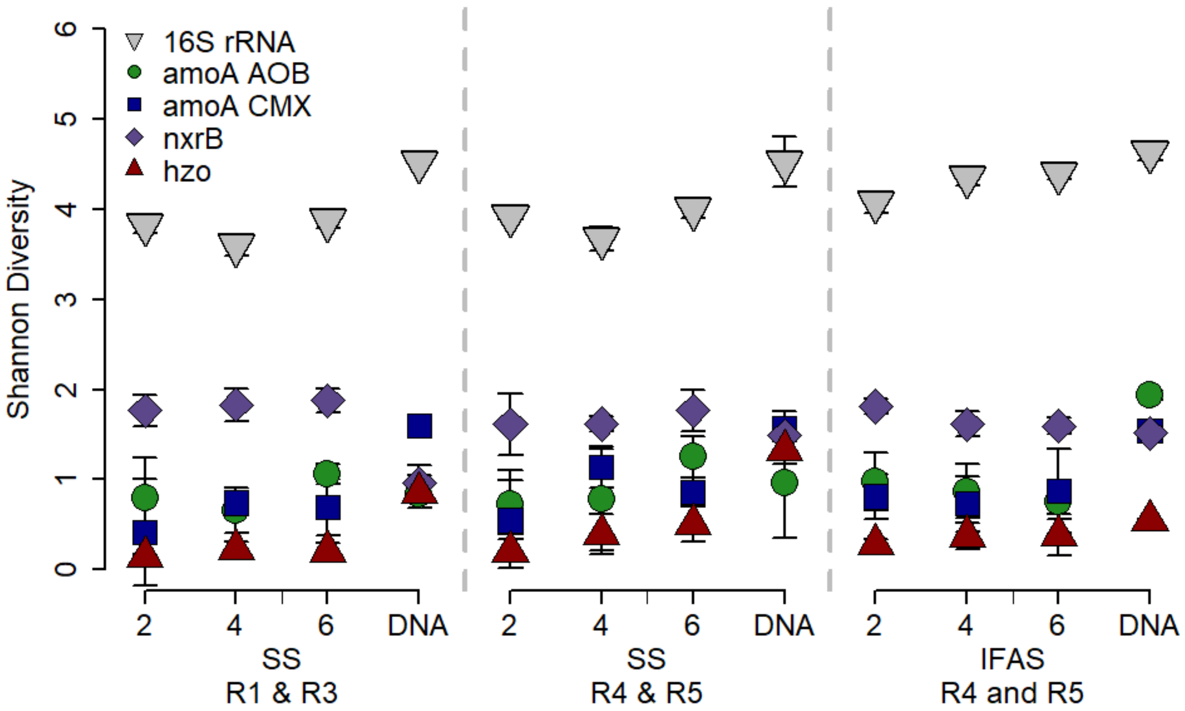

Figure S4: Shannon diversity indexes for the compositional analysis of 16S rRNA in gray inverted trianges, *amoA* for ammonia oxidizing bacteria in green circles, *amoA* for Comammox *Nitrospira* in blue squares, *nxrB* for nitrite oxidizing bacteria in purple diamonds, and *hzo* for anammox bacteria in red triangles.

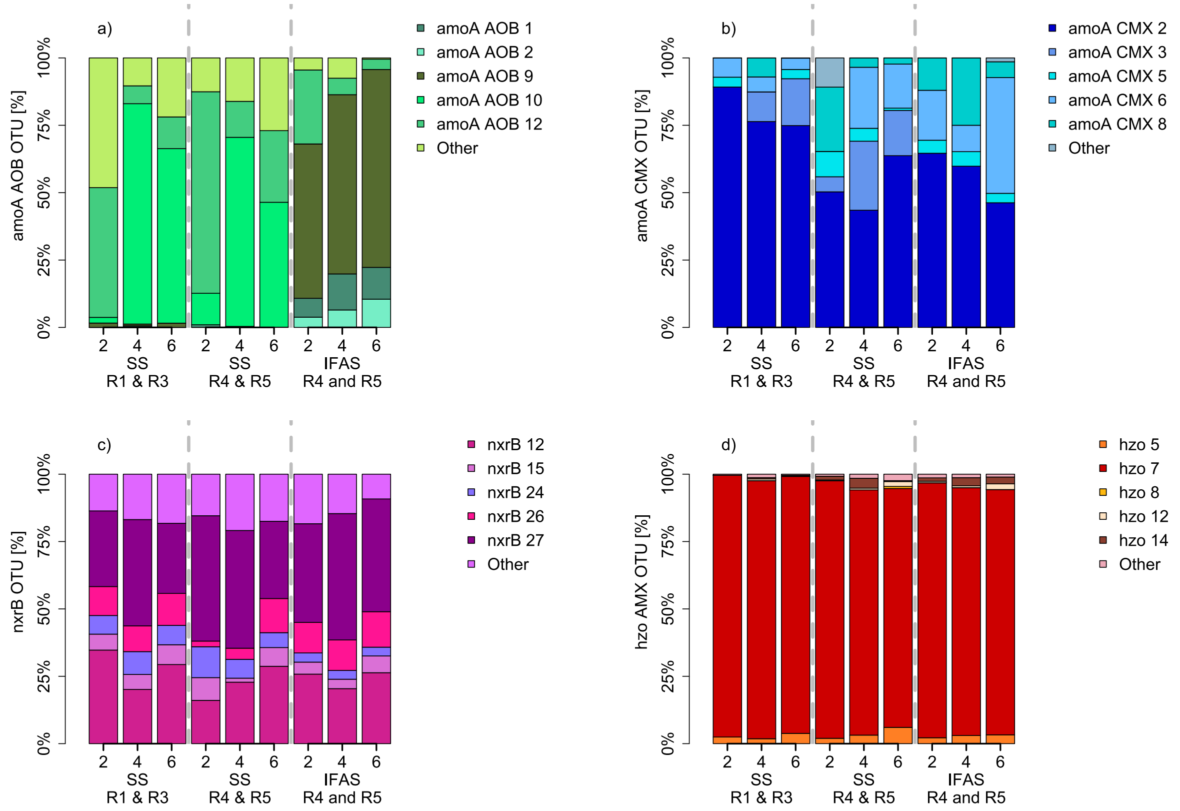

Figure S5: Composition of functional gene expression via amplicon transcript sequencing for a) *amoA* in strict ammonia oxidizing bacteria b) *amoA* in Commamox *Nitrospira* c) *nxrB* in all nitritie oxidizing bacteria, and d) *hzo* in anammox bacteria.

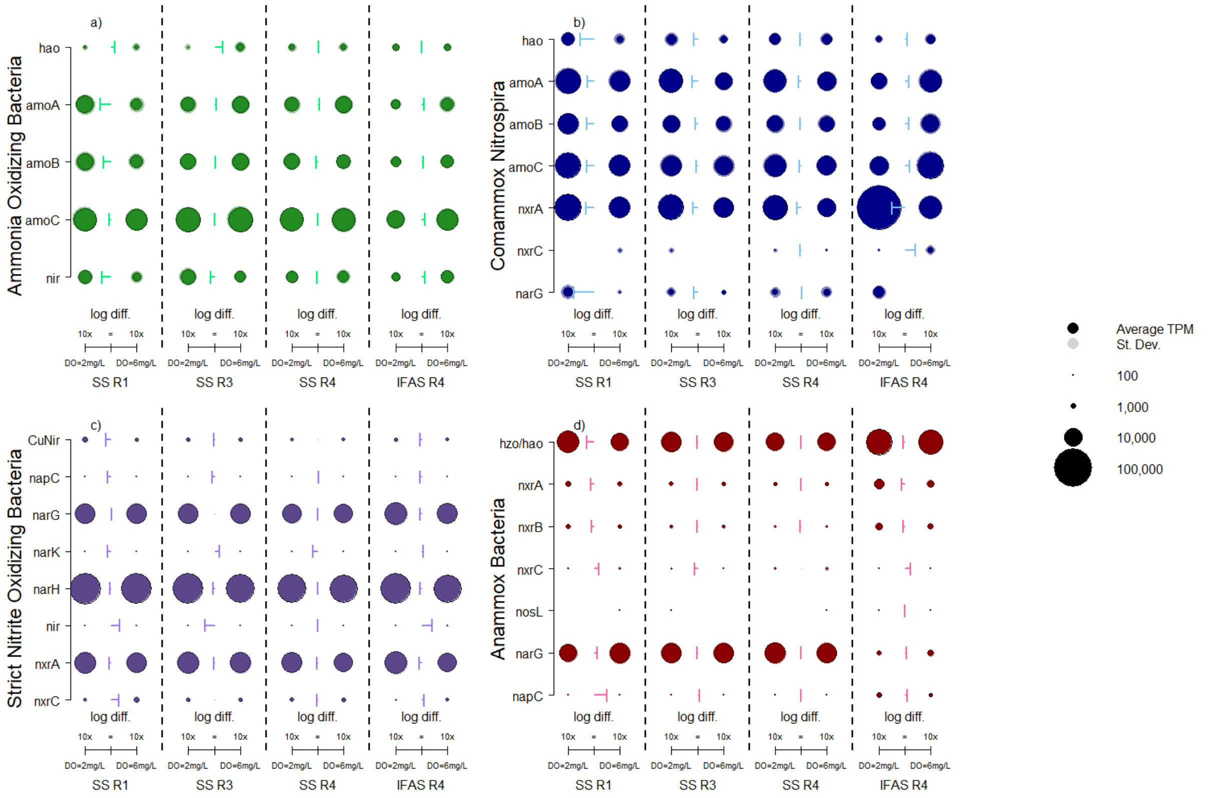

Figure S6: Transcripts-per-million activity profiles of major nitrogen cycling pathways mapped to MAGS constructed of a) strict ammonia oxidizing bacteria, b) comammox *Nitrospira,* c) strict nitrite oxidizing bacteria and d) anammox bacteria. The size of each point relates to the magnitude of transcripts-per-million, with an outline for standard deviation. The arrows show the magnitude difference between each data points DO = 2 mg/L and DO= 6 mg/L.

Text S1: Strict ammonia oxidizing bacteria and strict nitrite oxidizing bacteria dominate suspended sludge activity

Transcripts were mapped to previously assembled MAGS to determine the impacts of dissolved oxygen concentration on major nitrogen cycling pathways (**Supplemental Figure 6**). The nitrogen cycling pathways of ammonia oxidizing bacteria in suspended sludge were mostly unimpacted by dissolved oxygen concentration with nitrogen cycling transcripts comprising 3.76% ± 0.82% of all mapped AOB transcripts (**Supplemental Figure 6a**). The only exception was with *nir,* nitrite reductase, which significantly increased at low dissolved oxygen in R1 (Tukey p = 0.014). In IFAS at high dissolved oxygen concentrations, *amoC* and *nir* were significantly upregulated (Tukey $p_{amoC} = 0.002$, $p_{nir} = 0.001$). Overall nitrogen cycling expression in ammonia oxidizing bacteria did decrease in IFAS down to 1.77% ± 0.28% at DO = 2 mg/L and 3.04% ± 0.23% at DI = 6 mg/L.

Strict NOB were significantly more active at high dissolved oxygen concentrations in all systems (Tukey all $p << 0.05$) (**Supplemental Figure 6c)**. The percentage of nitrogen cycling transcripts mapped to the MAGS comprised 6.87% ± 0.63% in suspended sludge and 7.64% ± 0.68% in IFAS at DO = 2 mg/L, down to 6.24% ± 0.67% in suspended sludge and 5.70 ± 0.09% in IFAS at DO = 6 mg/L. Primarily, *nxrA,* and *narH* were most upregulated at low dissolved oxygen concentrations suggesting incomplete nitrogen removal causing an accumulation of nitrate and nitrite. While these two pathways did significantly increase at low dissolved oxygen concentrations, their largest increases were only 1.52x and 1.33x in IFAS respectively.
